# Atopic Dermatitis: Beyond the Skin and Into the Gut

**DOI:** 10.3390/jcm12175534

**Published:** 2023-08-25

**Authors:** Rachel L. Sadowsky, Pranvera Sulejmani, Peter A. Lio

**Affiliations:** 1 Rush Medical College, Rush University, Chicago, IL 60612, USA; rachel_l_sadowsky@rush.edu (R.L.S.); pranvera_sulejmani@rush.edu (P.S.); 2 Feinberg School of Medicine, Northwestern University, Chicago, IL 60611, USA; 3 Medical Dermatology Associates of Chicago, Chicago, IL 60654, USA

**Keywords:** atopic dermatitis, gut microbiome, gut–skin axis

## Abstract

Atopic dermatitis (AD) is a common, chronic and recurring inflammatory skin disorder characterized by an intensely pruritic, eczematous dermatitis. The etiology of AD is thought to involve a combination of environmental, genetic, and immunologic factors. Emerging research has investigated factors that may impact individual risk for developing AD, disease severity, and treatment response. One component is the gut microbiome, which is considered to play an essential role in maintaining the homeostasis of several organ systems. The gut microbiome has been described as a major regulator of the “gut–skin axis,” yet some studies have yielded conflicting evidence regarding the strength of the association of gut microbiota dysbiosis with AD. This review discusses recent insights into the role of the gut microbiome in AD pathogenesis and its interplay among other complex systems that govern the current assessments of and treatments for AD.

## 1. Introduction

Atopic dermatitis (AD) is a relapsing inflammatory disorder of the skin. It manifests as a result of genetic, immunologic, and environmental factors that disrupt the skin barrier and cause itching and inflammation [1]. Up to 20% of children and 5% of adults are affected by AD, and some studies have reported an increased prevalence in recent decades [1]. Potential factors that contribute to the development of AD include changes in the integrity and function of the epidermal barrier, disruptions in the skin microbiome, immune factors, allergens, bacterial and fungal infections, as well as the environment and genetics. While treatment for this condition has progressed significantly with the introduction of targeted immunomodulatory therapies, researching the links between the integumentary, digestive, nervous, and endocrine systems, as well as the mind, has the potential to improve our understanding of the disease and stimulate novel therapeutic modalities.

In the early 20th century, researchers began to recognize the importance of the human microbiome in maintaining health and preventing disease. The gut microbiome, defined by the microorganisms inhabiting the gastrointestinal tract, relies on a symbiotic balance with its host and closely communicates with nearby immune cells [2]. Its role in various health conditions has been investigated, and evidence supporting the association of allergic disease with changes in the gut microbiome is now abundant. Specifically, the gut microbiome is now considered to be a key regulator in the gut–skin axis, with recent studies demonstrating a potential relationship between changes in the gut microbiome and AD [3]. The continuous expansion of our understanding of the interaction of extracutaneous organ systems that impact the pathogenesis and disease course of AD has the potential to improve the quality of life for patients suffering from this chronic skin condition.

## 2. Materials and Methods

A comprehensive literature review was conducted on PubMed to isolate relevant studies investigating the role of the gut microbiome in the development of atopic dermatitis. Only studies written in English and providing insight into the relationship between the gut microbiome and the pathogenesis of atopic dermatitis were included in this review.

## 3. Skin Microbiome and AD

The Human Microbiome Project Consortium described the variability of microbial communities among all individuals and suggested that this variation can help to elucidate the links between the human microbiome and disease [4]. For example, the skin microbiota of healthy individuals and those with atopic dermatitis differ in their composition, with certain variations found more commonly in AD patients [5].

*Staphylococci*, particularly *Staphylococcus aureus* (*S. aureus*), are found in greater abundance on the skin of AD patients. Interestingly, chronic eczematous lesions have shown *S. aureus* greater abundance compared to acute lesional or non-lesional skin, indicating a correlation between *S. aureus* and disease severity [6]. Furthermore, a deficiency of the epidermal structural protein filaggrin, a result of a loss-of-function mutation seen in up to one-third of AD patients, may enhance the growth of *S. aureus* [3,7]. Studies comparing AD patients with positive *S. aureus* colonization to those without have also shown the latter to display significantly lower levels of some ceramides, triglycerides, and free fatty acids [8,9]. While *S. aureus* has been the most studied bacterium in AD patients, other species such as *S. epidermidis*, *S. haemolyticus*, *S. hominis*, and *S. lugdunensis* have also been shown to play a role in AD pathogenesis [6].

Low cutaneous microbial diversity is another common characteristic of atopic skin, and is even shared by affected and non-affected areas in patients with AD [3,10]. The inflamed skin of AD patients has been shown to have decreased abundance of *Cutibacterium*, *Streptococcus*, *Acinetobacter*, *Corynebacterium*, and *Prevotella* genera [3]. As a potential result, AD pathogenesis has also been linked to an alteration of epidermal lipid metabolism. Short-chain fatty acids (SCFAs), which have been shown to have an anti-inflammatory effect in the skin, are reduced in the lesional skin of AD patients. This is likely due to the decreased relative abundance of genera such as *Cutibacterium*, which normally produce SCFAs in healthy skin [3,11].

Studies suggest that the decreased overall diversity of skin microbiota and resultant increased abundance of *Staphylococci* correlated with AD disease severity may, accordingly, be associated with additional disease features, such as pruritus [12,13]. *S. aureus* colonization is thought to aggravate itching via protease-driven skin barrier disruption and skin pH alteration. As a result, *S. aureus* abundance has also been shown to correlate positively with factors related to itch intensity, including excoriations, loss of sleep, and subjective itch severity [6,14]. Given the recent advances in research elucidating deeper connections between AD pathogenesis and neuroimmunomodulation, some experts have looked to the gut for further evidence to support the balancing of microbiomes as a viable avenue for AD management.

## 4. Gut Microbiome and AD

The human gut is home to over 100 trillion microorganisms, and its ecosystem is highly complex and diverse [4]. The compositions and functions of the gut microbiota are affected by various factors, including genetics, age, diet, and medical treatments [6,15,16]. Starting in infancy, the acquisition of healthy gut flora contributes greatly to the development of the immune system. Disruptions or alterations in healthy gut flora may lead to dysbiosis, which has been linked to various conditions such as inflammatory bowel disease, obesity, and neuropsychiatric or allergic disorders [17].

The association between the dysbiosis of gut microbiota and AD has been previously described. Multiple studies have found that gut microbiota can influence resident cutaneous immune cells, forming what has become known as the “gut–skin axis” [6,18,19]. A 2020 study from Lee et al. found that patients with AD had a significantly different gut microbiota composition compared to healthy controls, with reduced microbial diversity and altered bacterial taxa [20]. Patients with AD were found to host a larger abundance of *Clostridium difficile*, *Escherichia coli*, and *S. aureus* in the gut and a lower proportion of *Bifidobacteria*, Bacteroidetes, and Bacteroides compared to healthy patients without AD [20].

Researchers have probed further into the underlying mechanisms leading to the development of AD in patients with gut dysbiosis. Increased intestinal permeability (i.e., a leaky gut) is more likely to be found in patients with gut dysbiosis. It has been shown to encourage toxin production and allow microorganisms and toxins to enter into circulation, ultimately impacting the skin [3,18,19]. Butyrate-producing bacteria found in the gut, including *Bifidobacterium*, *Lactobacillus*, *Clostridium*, *Bacteroides*, *Streptococcus*, and their metabolites, regulate the proliferation and activation of Treg cells and decrease the permeability of the intestinal epithelial barrier, thereby impacting inflammation in non-enteral organs, including the skin [21,22]. Nyland et al. found the abundance of these butyrate-producing microbes to be inversely correlated with AD disease severity among infants [21]. Furthermore, the Western diet has also been linked to gut dysbiosis and the development of AD. This is because a lack of anti-inflammatory SCFAs in the diet may alter the genes of gastrointestinal (GI) microbes and cause an inflammatory response in both the GI tract and the skin [23]. These shifts in gut microbial composition and abundance, and subsequent functional imbalances in AD patients, are deeply intertwined with complex immunological, neuroendocrine, metabolic, and psychological pathways [20].

## 5. Other Systems Involved in AD Pathogenesis

A broader relationship connecting inflammatory skin disorders with the immune and nervous systems was conceptualized in 1998 by O’Sullivan, et al., with the introduction of the neuro-immuno-cutaneous-endocrine (NICE) network [24]. The authors proposed a “tangled web” connecting the mind and body through common links between the central nervous system (CNS) and the skin, such as neuropeptides contained within cutaneous nerves that can induce properties in the skin associated with inflammation, including vasodilation, edema, and epithelial cell proliferation [24]. In AD, inflammatory cells and cytokines associated with a type 2 immune response are directly linked to pruritus, a defining characteristic of the disease [25]. Interleuken IL-31 was the first cytokine to be associated with itching in 2004, and since then it has been a target of interest in anti-itch research, including in the treatment of AD-associated pruritus [25]. 

**Figure 1:**
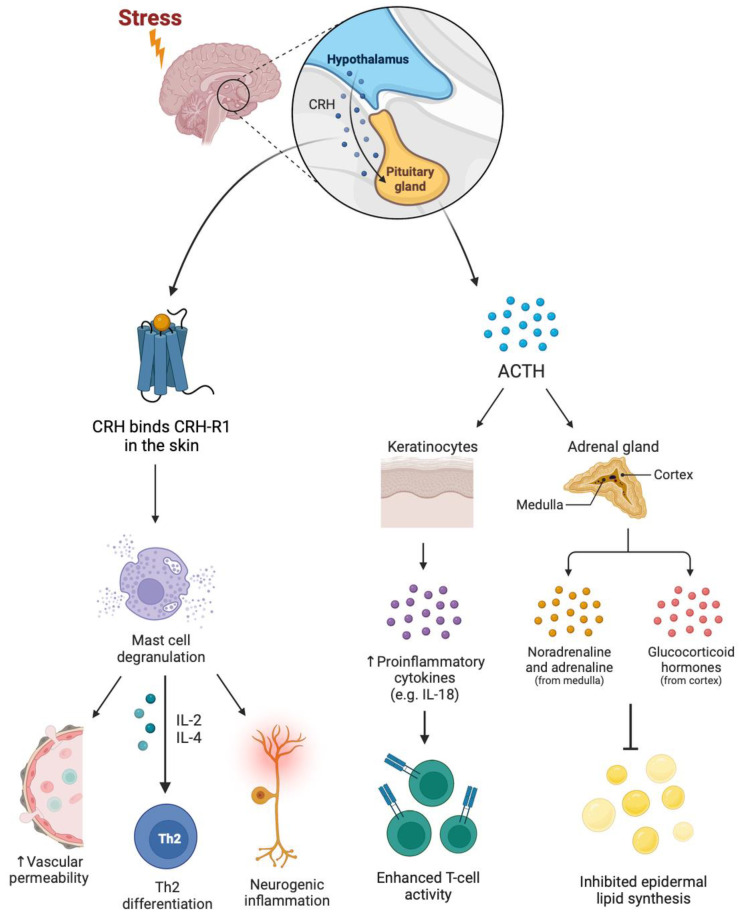
A model of stress causing the increased production of corticotropic releasing hormone (CRH) through the hypothalamic–pituitary–adrenal (HPA) axis, resulting in pro-inflammatory signaling. ACTH: Adrenocorticotropic Hormone.

The concept of communication among behavioral, neural, and immune functions has existed for decades [26]. An interest in the interaction of stress and other psychological processes with the nervous and immune systems and their combined impact on skin disease has also taken form. Stress is known to increase the production of corticotropic releasing hormone (CRH) through the hypothalamic–pituitary–adrenal (HPA) axis, with downstream effects on multiple pro-inflammatory intracellular signaling pathways [27] (See Figure 1).

In a study conducted by Buske-Kirschbaum et al., patients with AD were found to have a blunted HPA axis responsiveness with concomitant hyperactive sympathetic adrenomedullary systems. This is thought to potentially contribute to increased patient susceptibility to allergic inflammation [28]. More recently, evidence has shown that GI neuroendocrine molecules found in cutaneous nerves can also effect changes in brain function, anxiety, and stress that can ultimately modify gut epithelial permeability, and, as a result, affect the skin barrier function and cutaneous inflammatory response [20]. 

In 2010, Arck et al. introduced the “gut–brain–skin axis” in their exploration of the effect of microbiota modulation on stress-induced systemic and cutaneous inflammation [2,29]. GI peptides, such as calcitonin gene-related peptide and vasoactive intestinal peptide, have been detected within the fibers of cutaneous nerves [29]. These neuroendocrine molecules have been associated with the degree of AD symptom severity as they mediate pathways that disrupt the skin barrier and dysregulate the immune system [20]. The gut microbiome also produces substances such as tryptophan, which can contribute to skin itching, and γ-aminobutyric acid (GABA), which can inhibit itching sensations in the skin [20]. As a result, when the gut microbiome is in dysbiosis, these regulatory mechanisms of the gut–brain–skin axis are further altered, and the symptoms and severity of AD can be affected. As we continue to deepen our understanding of AD pathogenesis beyond the skin, it is important to consider the gut in conjunction with the nervous and immune systems when devising individualized treatment plans for patients.

## 6. Therapeutic Approaches

Holistic approaches to the medical management of conditions such as AD are increasingly sought. Traditionally, the management of AD has relied on symptomatic pruritus relief, phototherapy, topical medications, or immunomodulating agents such as dupilumab, tralokinumab, baricitinib, and upadacitinib. In patients with AD that is refractory to these first-line therapies, or perhaps in addition to them when desired, multimodal treatment regimens could be considered, including those targeting the gut–skin–brain axis.

The many metabolites and signal molecules produced by gut microbiota can affect an individual’s systemic immune response, making them potential targets for regulation [30]. Given the crucial role the gut microbiome has in the development and progression of AD, modulating it through prebiotics, probiotics, or postbiotics may hold promise as a therapeutic approach. One study described the potential therapeutic role of probiotics in increasing the synthesis of SCFAs in the gut and restoring the balance of gut microbiota through the use of mouse models [22,31]. Another study systematically reviewed large randomized controlled trials (RCTs), demonstrating the reduced incidence of AD in infants taking probiotics [32,33]. However, detailed protocols are needed to standardize the appropriate treatment parameters for probiotics in the prevention or treatment of AD [22].

Another field of research associated with AD and the gut microbiome is the potential benefit of fecal microbial transplantation (FMT), which is considered to be the most direct method of regulating gut dysbiosis [32]. A study by Mashiah et al. used the scoring atopic dermatitis (SCORAD) score to assess responses to FMT [34]. Despite a small cohort of four patients, FMT yielded significant improvement in signs and symptoms of AD without notable side effects. However, the practice of FMT has been noted to potentially result in severe complications and even death, and standardized treatment protocols are lacking [32].

The HPA axis and other cutaneous neuroendocrine pathways that are potentially linked to AD pathogenesis offer additional avenues for therapeutic guidance. For example, low-dose oral and topical naltrexone, a centrally acting opioid receptor antagonist, has shown antipruritic activity in AD patients [35]. Nalfurafine, a kappa opioid receptor agonist, has also been studied in mouse and monkey models of AD and induced reductions in both the pruritus and infiltration of CD4+ and CD8+ T-cells in the dermis [35]. A study has shown that the peripheral kappa opioid receptor agonist, difelikefalin, can improve itching and quality of life in patients with AD, and another study is evaluating it as an adjunctive treatment to topical corticosteroid in AD patients (NCT05387707) [32]. Further research is needed to confirm the efficacy and safety of these treatments in human subjects, but their potential to dampen AD symptoms at the very least holds promise.

Finally, the psychological factors connected with the neuroendocrine and cutaneous pathways involved in AD should also be considered as potential targets for treatment in this patient population [36]. Psychological treatment, known as mind–body therapy (MBT), includes practices such as meditation, biofeedback, hypnosis, acupuncture, and other relaxation methods [37]. Hypnotherapy was shown several decades ago to be beneficial for AD patients with refractory symptoms [38]. A recent RCT conducted in Germany revealed potential beneficial changes to patient-reported baseline itching intensity, disease severity, and disease-associated quality of life after undergoing hypnotherapy and treatment with an intermittent fasting program [39]. Another case series studying the use of an osteopathic manipulative treatment to relieve cervical muscle tension in 20 patients with moderate-to-severe AD on no other medicated topical or systemic treatments demonstrated overall improvement in the visual analog scale score for pruritus, and the total hospital anxiety and depression score was also notably reduced [40]. Addressing the psychological impact of AD as adjunctive disease treatment has shown benefit in multiple clinical trials and should be included as a method in multimodal disease management [38,39,40].

## 7. Conclusions

The cutaneous manifestation of atopic dermatitis is the result of complex interplay among multiple systems of the human body. When addressing the multidirectional relationships among the nervous, immune, endocrine, and cutaneous systems, the gut should also be considered as a potential player in AD pathogenesis and disease management. The connections between skin and gut microbiota and AD can influence both an individual’s likelihood to develop the disease and the severity of disease after onset. The resultant dysbiosis may also lead to downstream effects on the body’s neuroendocrine and stress responses. As AD is a common chronic disease, more studies are needed to further elucidate the relationships of these systems with AD and identify standardized, multimodal therapies with which to optimize disease treatment and improve patients’ quality of life.
